# Case Report: Successful endoscopic full-thickness resection of an irregular submucosal tumor of the esophagus with surface focal white patches: horseshoe-shaped leiomyoma

**DOI:** 10.3389/fonc.2025.1583882

**Published:** 2025-08-08

**Authors:** Lili Pan, Chuanfang Wang, Chong Zhang, Lijuan Fan, Ran Ma

**Affiliations:** ^1^ Department of Gastroenterology, Jining No. 1 People’s Hospital, Shandong, Jining, China; ^2^ Department of Pathology, Jining No.1 People’s Hospital, Shandong, Jining, China

**Keywords:** endoscopic full-thickness resection, irregular submucosal tumor of the esophagus, white patches, horseshoe-shaped leiomyoma, endoscopic ultrasound

## Abstract

Esophageal leiomyoma is a rare benign tumor of the esophagus, accounting for approximately 1.2% of all esophageal tumors. Endoscopically, it typically appears as a protrusion into the esophageal lumen with intact and smooth mucosa, which can be slightly moved with biopsy forceps and rarely causes luminal stenosis. The current case presents an irregular submucosal esophageal mass with localized white patches on the surface—an endoscopic appearance not previously reported. These surface white patches complicated the diagnosis, and the postoperative pathological finding of a horseshoe-shaped leiomyoma is even more unusual. Preoperative imaging with chest and abdominal CT and endoscopic ultrasound identified the lesion in the muscularis propria, and successful endoscopic full-thickness resection was performed. This minimally invasive approach was confirmed by pathology to be precise, safe, and effective, achieving treatment outcomes comparable to traditional surgical resection.

## Introduction

Esophageal Leiomyomatosis is a rare benign tumor, usually asymptomatic, with symptomatic tumors typically larger than 5 centimeters in diameter (1, 2). Although the exact cause is unclear, they are believed to be caused by local smooth muscle hyperplasia or genetic mutations (3). Esophageal leiomyomas are commonly found in patients aged 20–50 years, with 80% occurring in the middle to lower third of the esophagus (4). Pathologically, leiomyomas consist of interlacing bundles of smooth muscle cells and can occasionally grow to a diameter of up to 20 centimeters. A comprehensive review of over 800 cases reported in the world literature revealed only 2 cases (0.2%) showing malignant transformation from leiomyoma to leiomyosarcoma (5). There are reports (6) of esophageal leiomyoma accompanied by high-grade squamous intraepithelial neoplasia, suggesting a risk of malignant transformation in esophageal leiomyoma, with mechanisms involving the proliferation of mesenchymal cells (7). Therefore, particular attention should be given to lesions with superficial focal leukoplakia. Treatment for esophageal leiomyoma includes surgical resection, typically requiring esophagectomy and reconstruction. With the development of endoscopic resection techniques, submucosal tunneling with endoscopic removal (STER) and endoscopic submucosal excavation (ESE) are currently used by endoscopists to remove submucosal tumors (SMTs) of the gastrointestinal tract (8). With the development of endoscopic ultrasound and magnifying endoscopy, this case, based on the characteristics of esophageal leiomyoma, presented clear boundaries, was typically solitary and firm, and easy to dissect. The combination with CT was helpful in displaying the size and location of the tumor mass, suggesting the possibility of esophageal leiomyoma. In this case, correct diagnosis of a tumor in the muscularis propria, covered by focal leukoplakia, a very rare endoscopic finding, was made through thoracoabdominal CT and endoscopic ultrasound. We successfully performed endoscopic full-thickness resection (EFTR) to treat the lesion. The purpose of this case report is to deeply investigate the role of advanced endoscopic techniques in the diagnosis and treatment of esophageal leiomyoma, especially in cases involving concurrent superficial lesions, and to review current treatment strategies for this condition.

## Case report

**Figure 1:**
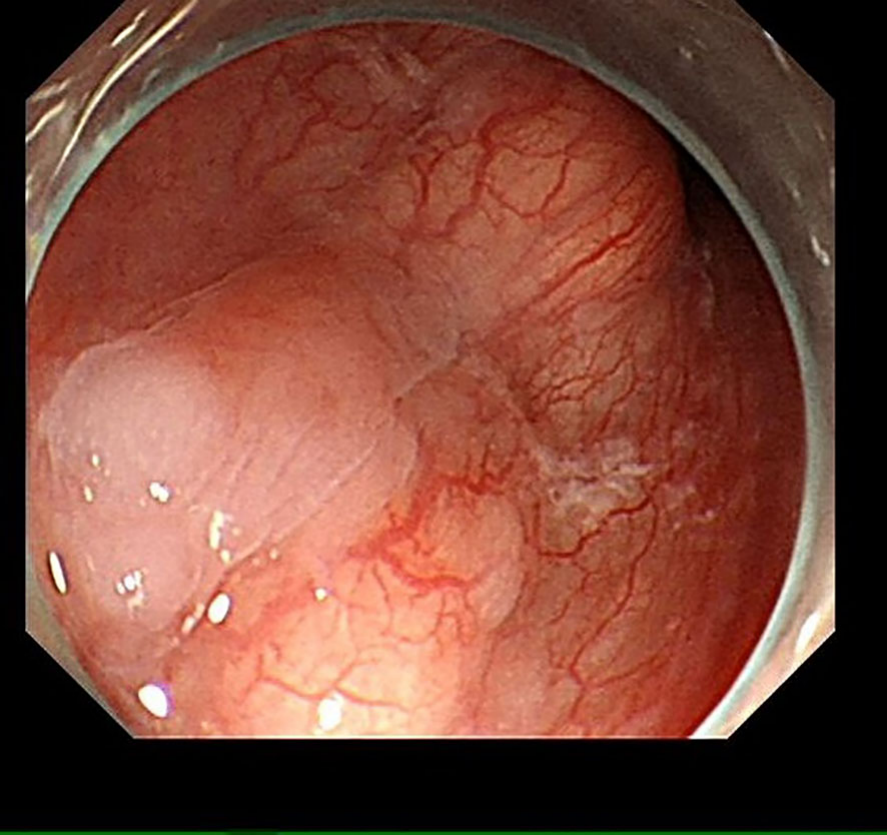
Under endoscopy, an irregular submucosal mass about 5 cm in size is visible on the left wall of the esophagus, with partial surface coverage of white patches.

**Figure 2:**
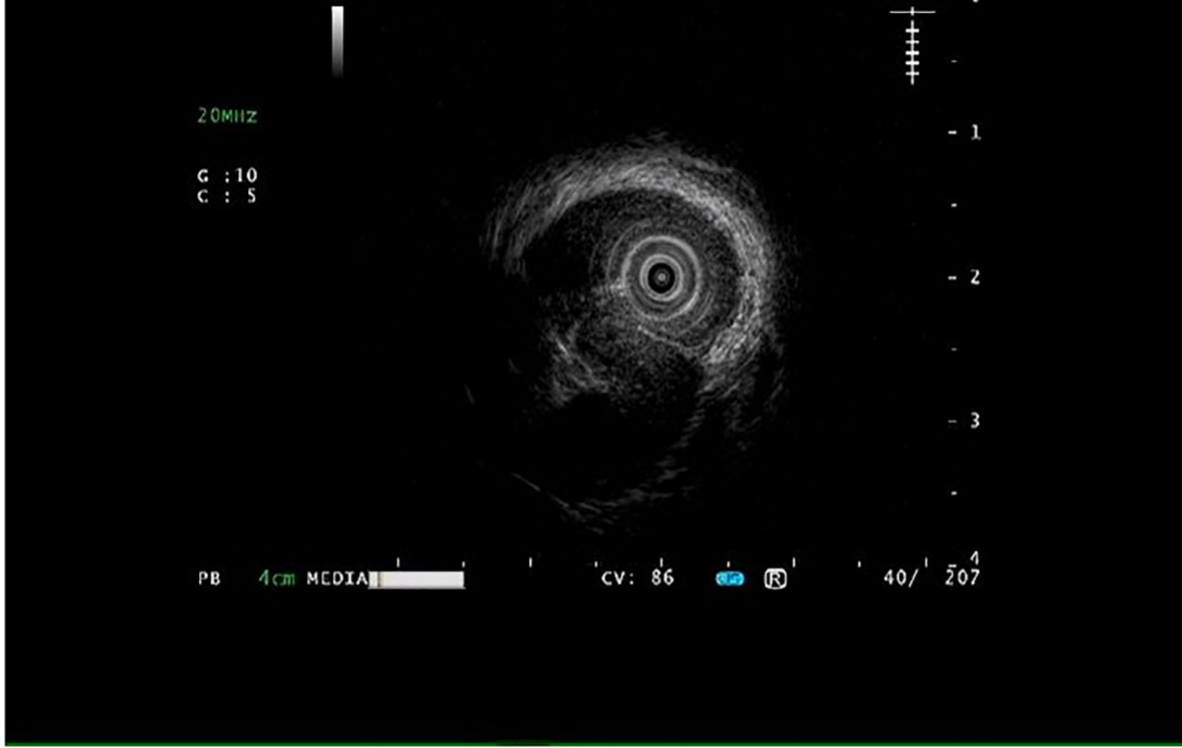
Endoscopic ultrasonography reveals that the structures of the first, second, and third layers are clear and intact, with a homogeneous hypoechoic area observed in the fourth layer.

A 55-year-old male was admitted to the hospital due to the discovery of a submucosal esophageal mass for over 8 months, with no prior health issues. The patient did not experience any discomfort such as dysphagia. A thorough enhanced CT scan of the chest and abdomen indicated local thickening of the lower esophageal wall, with no signs of tumor lesions or enlarged lymph nodes around the esophagus. Endoscopy revealed a longitudinally irregular submucosal mass with partial surface coverage of white patches (**Figure 1**), smooth in appearance, located approximately 34–39 cm from the incisors, showing a submucosal mass about 5 cm in size on the left wall of the lower esophagus. Endoscopic ultrasound findings: The first, second, and third layer structures were clear and intact, with a homogeneous hypoechoic area observed in the fourth layer, suggesting the lesion originated from the muscularis propria, with clear boundaries (**Figure 2**).

**Figure 3:**
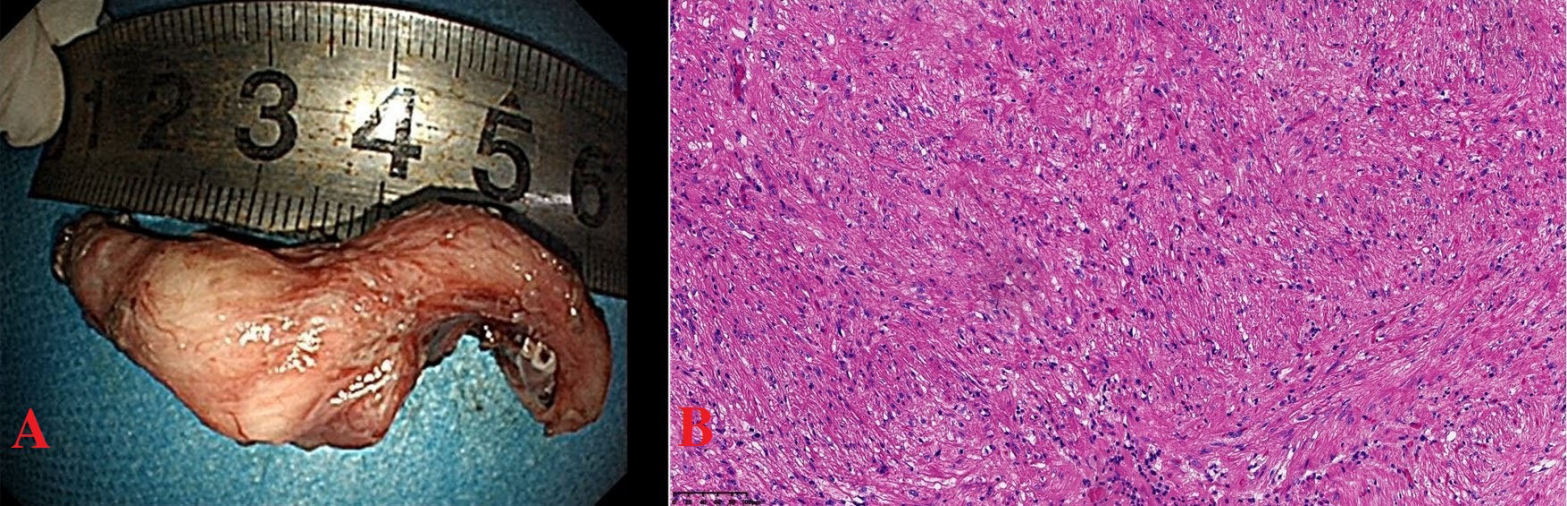
**(A)** Submucosal tumor, 5cmx2cm, horseshoe-shaped. **(B)** Tumor of long spindle cells, fascicles or woven, eosinophilic cytoplasm, elongated nuclei, clear boundaries, HE×200.

Informed consent was obtained from the patient for endoscopic full-thickness resection (EFTR). The lesion was marked around with a Dual knife, and the submucosa at the lesion site was injected with adrenaline methylene blue glycerin fructose injection, causing the mucosa to lift. The lesion was then cut and separated using a Dual knife and an IT knife, and the specimen was retrieved for examination. The submucosal tumor measured approximately 5cm (**Figure 3A**). The wound was treated with hemostatic forceps and then closed with 11 hemostatic clips. A three-lumen gastric tube was retained for 72 hours postoperatively.

The postoperative pathology showed that the tumor was located beneath the squamous epithelium and was mainly composed of long spindle cells arranged in fascicles or a woven pattern, with low density, eosinophilic cytoplasm, elongated nuclei, and a mild morphology. The boundaries were clear, and the diagnosis was esophageal leiomyoma (**Figure 3B**). No malignant lesions were found in the surface white patches. The immunohistochemical results were: SMA(+), Desmin(+), Ki67(+, 1%), P53 (wild-type staining pattern). In this patient, the immunohistochemical results showed positive expression of SMA and Desmin, which strongly supported the diagnosis of esophageal smooth muscle tumor. Postoperative pathology remains the gold standard for diagnosis. The pathological diagnosis of the tumor was leiomyoma. The patient recovered smoothly after the surgery.

## Discussion

In this case, the postoperative pathology revealed a rare horseshoe-shaped esophageal leiomyoma. The presence of localized leukoplakia on its surface added complexity to the diagnosis and increased the risk of clinical oversight or misdiagnosis. Based on thoracoabdominal CT and endoscopic ultrasound, the lesion appeared well-defined, suggesting a benign tumor. The diagnosis was ultimately confirmed through EFTR, and no malignant changes were found in the leukoplakic area. This case aligns with previous reports suggesting that esophageal leiomyomas may offer a degree of protection by preventing the spread and deep invasion of overlying squamous cell carcinoma (9, 10).

In this case, endoscopic full-thickness resection (EFTR) guided by endoscopic ultrasound was used to treat esophageal submucosal tumors with localized leukoplakia. The results demonstrated that this method is characterized by high precision, minimally invasive nature, safety and effectiveness. EFTR achieves comparable therapeutic effects to surgical procedures by completely removing the entire layer of the lesion. However, it causes less trauma and enables patients to recover more quickly. With the development of endoscopic resection techniques, submucosal tunnel endoscopic resection (STER) and endoscopic dissection resection (ESE) have now been widely used for the removal of gastrointestinal submucosal tumors (8). However, secure closure remains the main technical challenge in the management of iatrogenic perforations during EFTR. In recent years, the double-channel endoscopic purse-string suturing is an effective and safe method for closing large perforations (11). However, the parallel design of the two channels can make the coordination of instruments challenging, especially when grasping the nylon ring around the perforation margins (12). In addition, over-the-scope clip (OTSC) systems, endoscopic suturing devices, and dual-channel endoscopes are not routinely available in most endoscopy units in China, which limits their application and the operator’s experience. In our case, after completing the EFTR procedure, we used hemostatic forceps to treat the wound and closed the incision one by one with 11 hemostatic clips. After the operation, the patient was instructed to fast for 72 hours. Combined with proton pump inhibitor therapy, intravenous fluid support and the insertion of a three-lumen gastric tube, the aim was to reduce the incidence of postoperative complications, promote wound healing and shorten the hospital stay. This case provided new clinical experience and ideas for the innovative treatment strategies and postoperative care of EFTR.

## Conclusion

This case describes a rare horseshoe-shaped esophageal leiomyoma with surface white patches. After comprehensive preoperative assessment with imaging and endoscopy, the lesion was successfully removed by endoscopic full-thickness resection. Postoperative pathology confirmed a benign leiomyoma with no malignant changes in the white patch area. This case highlights the importance of thorough evaluation for submucosal esophageal tumors with surface leukoplakia and supports endoscopic full-thickness resection as a safe, effective, and minimally invasive treatment option with clinical value.

## Data Availability

The original contributions presented in the study are included in the article/supplementary material. Further inquiries can be directed to the corresponding authors.
